# The Effects of a Whey Protein and Guar Gum-Containing Preload on Gastric Emptying, Glycaemia, Small Intestinal Absorption and Blood Pressure in Healthy Older Subjects

**DOI:** 10.3390/nu11112666

**Published:** 2019-11-05

**Authors:** Hung Pham, Iselin S. Holen, Liza K. Phillips, Seva Hatzinikolas, Lian Q. Huynh, Tongzhi Wu, Trygve Hausken, Christopher K. Rayner, Michael Horowitz, Karen L. Jones

**Affiliations:** 1 Centre of Research Excellence in Translating Nutritional Science to Good Health, Adelaide Medical School, The University of Adelaide, Adelaide 5000, Australia; hung.pham@adelaide.edu.au (H.P.); liza.phillips@adelaide.edu.au (L.K.P.); seva.hatzinikolas@adelaide.edu.au (S.H.); lian.huynh@student.adelaide.edu.au (L.Q.H.); tongzhi.wu@adelaide.edu.au (T.W.); chris.rayner@adelaide.edu.au (C.K.R.); michael.horowitz@adelaide.edu.au (M.H.); 2 Department of Clinical Medicine, University of Bergen, 5021 Bergen, Norway; iselinsh_93@hotmail.com (I.S.H.); trygve.hausken@helse-bergen.no (T.H.); 3 Endocrine and Metabolic Unit, Royal Adelaide Hospital, Adelaide 5000, Australia; 4 Department of Gastroenterology and Hepatology, Royal Adelaide Hospital, Adelaide 5000, Australia

**Keywords:** gastric emptying, guar gum, postprandial glycaemia, postprandial hypotension, preload, whey protein

## Abstract

A whey protein/guar gum preload reduces postprandial glycaemia in type 2 diabetes through slowing gastric emptying. However, gastric emptying has previously been assessed using a stable isotope breath test technique, which cannot discriminate between slowing of gastric emptying and small intestinal absorption. This preload also may be useful in the management of postprandial hypotension. We evaluated the effects of a whey protein/guar preload on gastric emptying, glucose absorption, glycaemic/insulinaemic and blood pressure (BP) responses to an oral glucose load. Eighteen healthy older participants underwent measurements of gastric emptying (scintigraphy), plasma glucose and insulin, glucose absorption, superior mesenteric artery (SMA) flow, BP and heart rate (HR) after ingesting a 50 g glucose drink, with or without the preload. The preload reduced plasma glucose (*p* = 0.02) and serum 3-O-methylglucose (3-OMG) (*p* = 0.003), and increased plasma insulin (*p* = 0.03). There was no difference in gastric emptying or BP between the two days. The reduction in plasma glucose on the preload day was related to the reduction in glucose absorption (r = 0.71, *p* = 0.002). In conclusion, the glucose-lowering effect of the preload may relate to delayed small intestinal glucose absorption and insulin stimulation, rather than slowing of gastric emptying.

## 1. Introduction

The use of a macronutrient preload given 15–30 min before a main meal—to diminish postprandial glycaemic excursions, represents a novel approach to manage type 2 diabetes (T2D) [1,2,3,4]. The underlying rationale is that the nutrients in the preload will stimulate the release of gut hormones, including glucagon-like peptide-1 (GLP-1), to augment insulin secretion and slow gastric emptying of the subsequent meal [5]. Gastric emptying, which is highly reproducible in a given individual but exhibits a substantial inter-individual variation in health [6], is now recognised to be normal, or modestly accelerated, in the majority of people with uncomplicated T2D and relatively good glycaemic control [7]. In this group, slowing of gastric emptying is associated with a lowering in postprandial glucose [7], which is the major contributor to average glycaemic control, which can be assessed by HbA1c [7]. We have recently evaluated a preload containing whey (17 g) and guar (5 g), added to a ‘shake and take’ cup containing 150 mL water in participants with T2D [2]. Whey protein (a by-product in the production of cheese) and guar gum (a viscous, soluble polysaccharide, commonly used as a food thickener) have both been associated with the slowing of gastric emptying [2,4]. In those studies, the preload was well-tolerated when taken twice a day and substantially reduced the blood glucose response to a mashed potato meal, and modestly decreased glycated haemoglobin (0.1%) in T2D patients who were already well-controlled (mean HbA1c 6.6  ±  0.1%), without inducing weight gain [2]. A subsequent study suggested that these effects were mediated predominantly by the whey protein; guar, in a dose of 5 g, was less effective for lowering postprandial glucose compared with 17 g whey [4]. In both studies, the decrease in postprandial glucose tended to be associated with a reduction in plasma insulin. Gastric emptying was assessed using a validated breath test technique in both studies, and reported to be slowed by the preload after both acute and 12 weeks’ administration [2,4]. However, a fundamental limitation of the breath test is that it is indirect; i.e., it cannot discriminate between effects on gastric emptying from those upon absorption of glucose in the small intestine, unlike the ‘gold standard’ technique of scintigraphy [8]; changes in small intestinal motility/absorption have been shown to have a major effect on postprandial glycaemia [9]. Accordingly, the effect of this preload on gastric emptying remains uncertain.

Preloads, including the whey/guar combination, also have potential to mitigate postprandial hypotension (PPH)—a reduction in systolic blood pressure (BP) of greater than 20 mmHg after a meal, a condition which is associated with falls and syncope [10,11], as well as increased mortality [12]. PPH currently lacks an effective treatment and occurs in 25%–40% of patients with T2D [11,13] and ~15% of the ‘healthy’ elderly [13,14]. We have shown that the fall in BP after a meal is greater when the rate of gastric emptying is faster in both healthy older subjects [14,15] and patients with T2D [16], while slowing gastric emptying with guar gum attenuates the fall [17,18]. Moreover, exogenous administration of GLP-1 [19] and the ‘short-acting’ GLP-1 receptor agonist, lixisenatide [20], both slow gastric emptying, and markedly attenuate the magnitude of the postprandial fall in BP. Accordingly, a whey/guar preload has the potential to represent a simple, safe and inexpensive approach in the management of PPH.

In this study, we aimed to determine the effects of the whey/guar preload on gastric emptying (measured with scintigraphy), intestinal glucose absorption and the glycaemic and BP responses to an oral glucose load in healthy older subjects.

## 2. Materials and Methods

### 2.1. Subjects

Eighteen healthy older subjects (7 male and 11 female, mean age 72.6 ± 1.1 years, BMI 26.3 ± 0.5 kg/m^2^) participated in the study. All were non-smokers and none had a history of gastrointestinal disease or surgery, diabetes, significant respiratory, renal or cardiac disease, chronic alcohol abuse, epilepsy or were taking medication known to influence BP or gastrointestinal function.

The study was registered at http://www.ANZCTR.org.au (ACTRN12619000438156), conducted in accordance with the Declaration of Helsinki, and approved by the Human Research Ethics Committee of the Central Adelaide Local Health Network (CALHN) (HREC/18/CALHN/197). Each participant provided written, informed consent prior to their involvement in the study.

### 2.2. Protocol

Each participant was studied on 2 occasions, separated by at least 7 days, in a randomised, crossover design. Randomisation was performed at the beginning of the first visit of each participant by an investigator tossing a coin. On each study day, participants attended the Clinical Research Facility, Adelaide Health and Medical Sciences building at The University of Adelaide at 09.00 after an overnight fast (14 h for solids; 12 h for liquids) [21]. They were seated with their back against a gamma camera (Siemens e.cam single-head gamma camera, Siemens Medical Solutions USA Inc, Knoxville, TN, USA) and an intravenous cannula was inserted into an antecubital vein for blood sampling. An automated BP cuff was placed around the opposite arm. Following a ‘rest period’ of 15–30 min, to stabilise the baseline BP [22], each participant was given either (i) a test drink containing 50 g glucose and 5 g 3-O-methylglucose (3-OMG)—an analogue of glucose that is not metabolised—for the assessment of small intestinal glucose absorption, made up to 300 mL with water and radiolabelled with 20 MBq ^99m^Tc-calcium phytate to assess gastric emptying using scintigraphy [23] (the ‘control’ day); or (ii) a ‘preload’ containing 16.4 g whey protein and 4.4 g guar (90 kcal) (Vanilla flavour-GlucoControlTM, Faulding; Omni Innovation, Campbellfield, VIC, Australia) made up to 150 mL with water 15 min before the test drink (the ‘preload’ day). The preload and drink were both consumed within 2 min. Time zero (*t* = 0 min) was defined as the time of drink completion.

Measurements of gastric emptying, plasma glucose, plasma insulin, serum 3-OMG, superior mesenteric artery (SMA) blood flow, BP and heart rate (HR) and were obtained until *t* = 120 min, given that in healthy subjects, blood glucose concentrations rise approximately 10 min after ingestion of a carbohydrate-containing meal to usually achieve a maximum value within the first hour [24] and PPH often occurs within 2 h following a meal [11]. At the end of each study day, participants were offered a light lunch before they left the laboratory.

### 2.3. Measurements

#### 2.3.1. Gastric Emptying

Radioisotopic data were acquired in 1-min frames for the first 60 min and at 3-min frames thereafter up to 120 min. A region-of-interest was drawn around the total stomach and data were corrected for participant movement, radionuclide decay and γ-ray attenuation [16,25]. Gastric emptying curves (representing the percentage of the maximum total stomach content over time) were derived, and the intragastric content at *t*  =  0, 15, 30, 45, 60, 90 and 120 min and the 50% emptying time (T50) were calculated [26].

#### 2.3.2. Plasma Glucose and Insulin

Venous blood samples (~10 mL) were obtained immediately prior to the ingestion of the preload (*t* = −18 min) and/or drink (*t* = −3 min) and then at *t* = 15, 30, 45, 60, 90 and 120 min. Samples were collected into ice-chilled EDTA-treated tubes and plasma was separated by centrifugation at 1996× *g* for 15 min at 4 °C within 15 min of collection and stored at −80 °C until assayed [27].

Plasma glucose was measured using the hexokinase technique (2900D Biochemistry Analyzer, YSI Incorporated, Yellow Springs, OH, USA) [27]. Plasma insulin was measured by ELISA (Diagnostics 10-1113, Mercodia, Uppsala, Sweden). The sensitivity of the assay is 1.0 mU/L and intra and inter-assay coefficients of variation are 2.9% and 6.7%, respectively [27].

The insulinogenic index (the ratio of change in insulin over change in glucose over the first 30 min after the glucose drink) was calculated to estimate early-phase insulin secretion [28].

#### 2.3.3. Oral Glucose Absorption (Serum 3-OMG)

Venous blood samples (~5 mL) were collected into untreated tubes and allowed to clot. Serum was separated by centrifugation at 1996× *g* for 15 min at 4 °C and stored at −80 °C until assayed [27]. Serum 3-OMG concentrations were measured by liquid chromatography and mass spectrometry, with a sensitivity of 0.0103 mmol/L [29].

#### 2.3.4. Superior Mesenteric Artery Blood Flow

Superior mesenteric artery (SMA) blood flow (mL/min) was measured immediately prior to the ingestion of the preload (*t* = −18 min) and/or drink (*t* = −3 min), and then at *t* = 15, 30, 45, 60, 90 and 120 min using a Logiq e ultrasound system with a 3.5C broad-spectrum 2.5–4 MHz convex linear array transducer (GE Healthcare Technologies, Sydney, Australia) [30].

#### 2.3.5. Blood Pressure and Heart Rate

Systolic and diastolic BP (SBP and DBP) and HR were measured with an automated oscillometric BP monitor (DINAMAP ProCare 100, GE Medical Systems, Milwaukee, WI, USA) at 3-min intervals prior to administration of the drink, and then at 5-min intervals from *t* = 0–120 min. Fasting BP and HR were calculated as an average of measurements obtained at *t* = −24, −21 and −18 min prior to the ingestion of the preload (*t* = −17 min), or at *t* = −9, −6 and −3 min prior to the ingestion of the drink (*t* = −2 min) on the study day without a preload. PPH was defined as a fall in SBP ≥ 20 mmHg that was sustained for ≥30 min [16].

#### 2.3.6. Cardiovascular Autonomic Nerve Dysfunction

Standardised cardiovascular reflex tests were used to assess autonomic nerve function [31,32,33]. Parasympathetic function was evaluated by the variation (RR interval) of HR during deep breathing and the immediate response to standing (“30:15” ratio). Sympathetic function was assessed by the decrease in SBP in response to standing. Each result was scored according to age-adjusted predefined criteria: 0  =  normal, 1  =  borderline and 2  =  abnormal, for a maximum total score of 6. A score  ≥  3 was considered to indicate autonomic dysfunction [33].

### 2.4. Statistical Analysis

Sample size requirements were determined via power calculations alpha = 0.05 and beta = 0.8, performed with systolic BP as the primary outcome parameter, based on previous data. Gastric emptying, plasma glucose and insulin, serum 3-OMG, SMA blood flow, SBP, DBP and HR were analysed and presented as absolute values. Two-way, repeated-measures analyses of variances (ANOVAs) with treatment and time as factors and Bonferroni’s correction for post hoc comparisons, were used to analyse gastric emptying, plasma glucose, plasma insulin, serum 3-OMG and SMA blood flow. One-way, repeated-measures ANOVA was used to evaluate the effect of time on SBP, DBP and HR. The maximum falls in SBP and DBP and increases in HR, plasma glucose and insulin, serum 3-OMG and SMA blood flow were defined as the greatest change from *t* = −3 min in each subject at any given time point on each study day. Areas under the curves (AUCs) for SBP, DBP, HR and serum 3-OMG were calculated. Incremental areas under the curves (iAUCs) were calculated for plasma glucose and insulin. AUCs for SBP, DBP and HR, the insulinogenic index and maximum increases/falls in the two study days were compared using Student’s paired *t*-tests. Pearson’s correlations were used to evaluate relationships between plasma glucose, plasma insulin and serum 3-OMG between the two study days. All analyses were performed using SPSS version 24 (SPSS, Chicago, IL, USA); a *p*-value < 0.05 was considered significant. Data are shown as mean values ± SEMs.

## 3. Results

The studies were generally well tolerated. No participant had autonomic nerve dysfunction. In one participant, extensive small intestinal overlap precluded acceptable gastric emptying analysis and the scintigraphic data were not included. In another subject, 3-OMG was inadvertently not included in the drink on one of the two days. Accordingly, paired BP, HR, SMA blood flow, plasma glucose and insulin data were available in 18 subjects, while gastric emptying and serum 3-OMG data were available in 17 subjects.

### 3.1. Gastric Emptying

**Figure 1:**
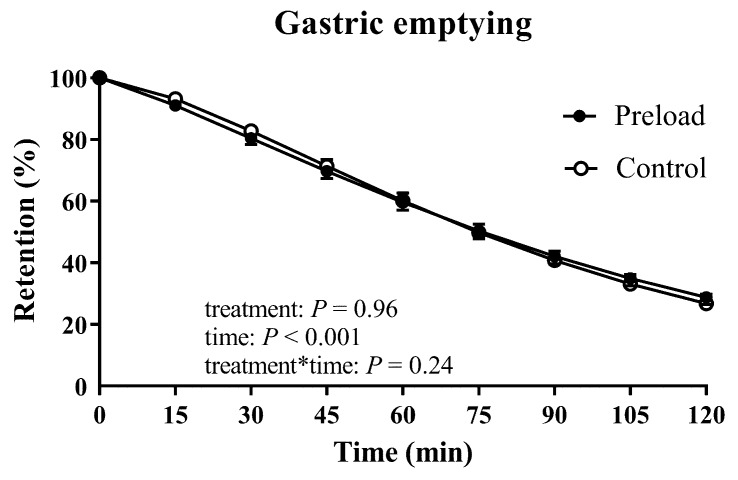
Retention in the total stomach, following ingestion of 50 g glucose in 300 mL water, on the control and preload days. Results of repeated measures ANOVA are reported as *p*-values for differences by treatment (treatment), differences over time (time) and differences due to the interaction of treatment and time (treatment × time). Data are means ± SEM (*n* = 17).

Gastric emptying of the glucose drink followed an overall linear pattern (time effect: *p* < 0.001, Figure 1). There was no difference in gastric retention evaluated by ANOVA (treatment effect: *p* = 0.96; treatment × time interaction: *p* = 0.24; Figure 1), nor T50 (preload: 76.5 ± 4.3 versus control: 76.5 ± 4.8 min, *p* = 0.99). At *t* = 120 min, gastric emptying was incomplete in all subjects on both days.

### 3.2. Plasma Glucose and Insulin

There were no differences in baseline (fasting) plasma glucose or insulin between the two days (Table 1). After ingestion of the preload, there was no change in plasma glucose between *t* = −18 min and *t* = −3 min (5.2 ± 0.1 versus 5.0 ± 0.2 mmol/L, *p* = 0.26), while plasma insulin increased (5.3 ± 0.8 versus 7.0 ± 1.1 mU/L, *p* = 0.02).

**Figure 2:**
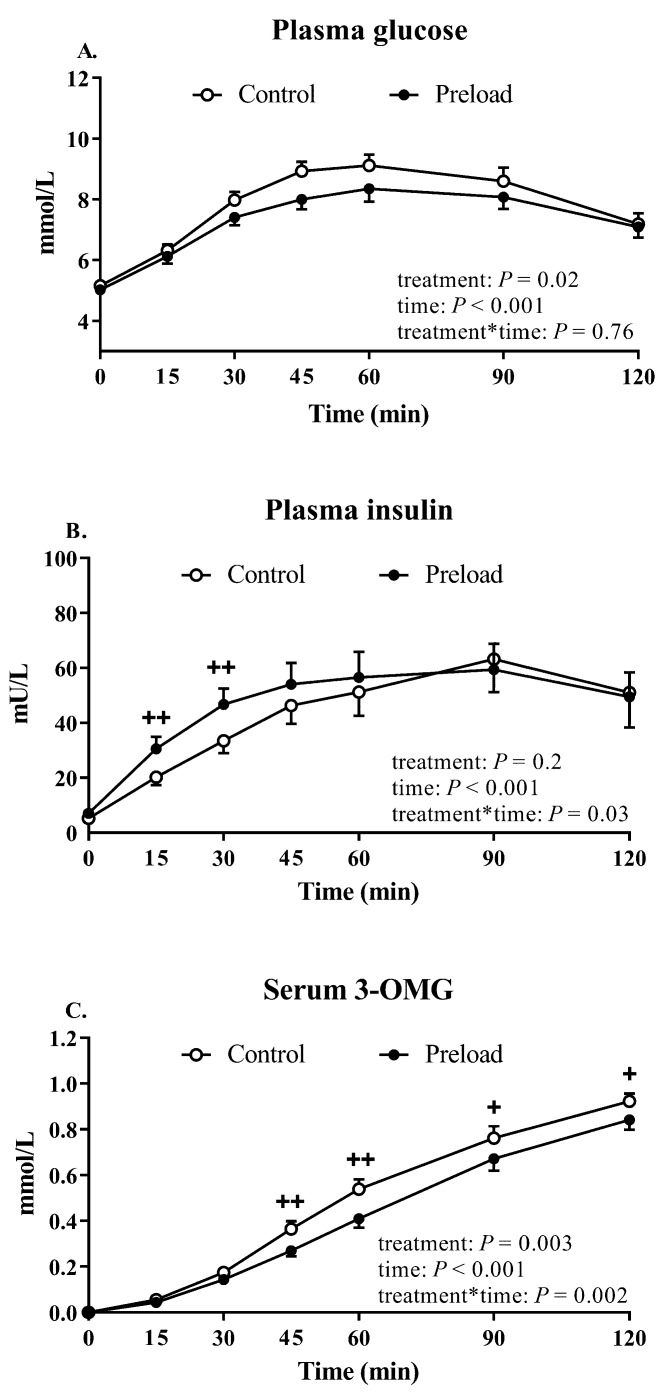
Plasma glucose (**A**), plasma insulin (**B**) and serum 3-OMG (**C**) on the control and preload days. Results of ANOVA are reported as *p*-values for differences by treatment (treatment), differences over time (time) and differences due to the interaction of treatment and time (treatment × time). Post hoc comparisons, adjusted by Bonferroni’s correction, were performed if ANOVA values (treatment × time) were significant. + *p* < 0.05 and ++ *p* < 0.01. Data are mean values ± SEM (*n* = 18 for plasma glucose and insulin and *n* = 17 for 3-OMG).

There was a rise in plasma glucose on both days following the glucose drink (time effect: *p* < 0.001, Figure 2A). Plasma glucose concentrations were lower after the preload compared to control (treatment effect: *p* = 0.02), with significant differences at *t* = 30, 45 and 60 min (*p* < 0.05 for each, Figure 2A). The maximum rise in plasma glucose after the glucose drink was also less after the preload (preload: 3.7 ± 0.4 versus control: 4.3 ± 0.3 mmol/L, *p* = 0.007).

There was a rise in plasma insulin on both days following the glucose drink (time effect: *p* < 0.001, Figure 2B). Plasma insulin concentrations were greater after the preload (treatment × time interaction: *p* = 0.03) at *t* = 15 and 30 min (*p* < 0.001 for each, Figure 2B). There was no difference in the maximum rise in plasma insulin after the glucose drink between the two days (preload: 63.1 ± 8.5 versus control: 62.8 ± 11.8 mU/L, *p* = 0.97).

The insulinogenic index at 30 min was higher after the preload, compared with control (preload: 18.1 ± 2.4 versus control: 10.9 ± 1.5, *p* = 0.002).

### 3.3. Glucose Absorption

There was an increase in serum 3-OMG on both the preload and control study days following the glucose drink (time effect: *p* < 0.001, Figure 2B). Serum 3-OMG concentrations were lower after the preload (treatment effect: *p* = 0.003 and treatment × time interaction: *p* = 0.002) with significant differences at *t* = 45, 60, 90 and 120 min (*p* < 0.05 for each, Figure 2C). The peak serum 3-OMG concentrations were also less after the preload (preload: 0.84 ± 0.04 versus control: 0.92 ± 0.03 mmol/L, *p* = 0.01).

### 3.4. Superior Mesenteric Artery Blood Flow

There was no difference in baseline (fasting) SMA blood flow between the two study days (Table 1). Following the preload, there was no change in SMA blood flow between *t* = −17 min to *t* = −2 min (329 ± 24.8 versus 371 ± 27.5 mL/min, *p* = 0.10).

**Figure 3:**
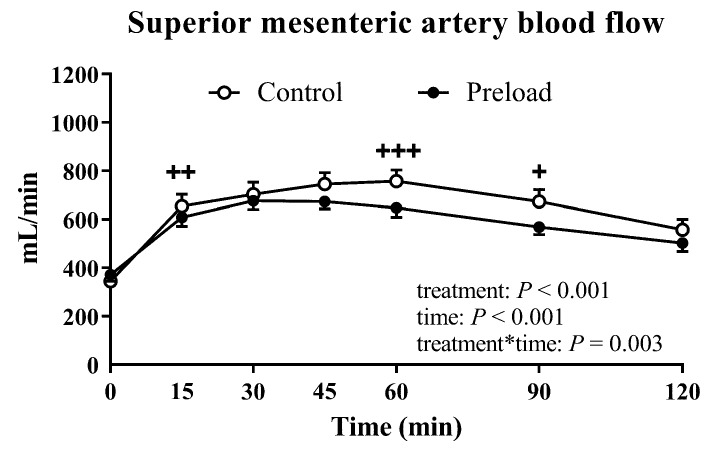
Superior mesenteric artery blood flow on the control and preload days. Results of ANOVA are reported as *p*-values for differences by treatment (treatment), differences over time (time) and differences due to the interaction of treatment and time (treatment*time). Post hoc comparisons, adjusted by Bonferroni’s correction, were performed if ANOVA values (treatment × time) were significant. + *p* < 0.05, ++ *p* < 0.01, +++ *p* < 0.001. Data are mean values ± SEM (*n* = 18).

There was an increase in SMA blood flow on both days following the glucose test drink (time effect: *p* < 0.001 for all, Figure 3). This rise was attenuated after the preload (treatment effect: *p* < 0.001 and treatment × time interaction: *p* = 0.003), with significant differences at *t* = 15, 60 and 90 min (*p* < 0.05 for each, Figure 3). The maximum increase in SMA flow was also less following the preload (preload: 365.5 ± 29.4 versus control: 488.2 ± 42.5 mL/min, *p* = 0.002).

### 3.5. Blood Pressure and Heart Rate

There were no differences in baseline (fasting) SBP, DBP or HR between the two days (Table 1). Following ingestion of the preload, there was a trend for an increase in SBP (120.0 ± 3.2 versus 124.3 ± 3.9 mmHg, *p* = 0.06), with no change in DBP (67.3 ± 1.9 versus 69.2± 2.1 mmHg, *p* = 0.22) or HR (63.1 ± 2.3 versus 63.1 ± 2.4 bpm, *p* = 0.17) from *t* = −18 min to *t* = −3 min. No participant had PPH, i.e., a sustained fall in SBP of more than 20 mmHg for 30 min, compared to their fasting measurement on either day.

#### 3.5.1. Systolic Blood Pressure

**Figure 4:**
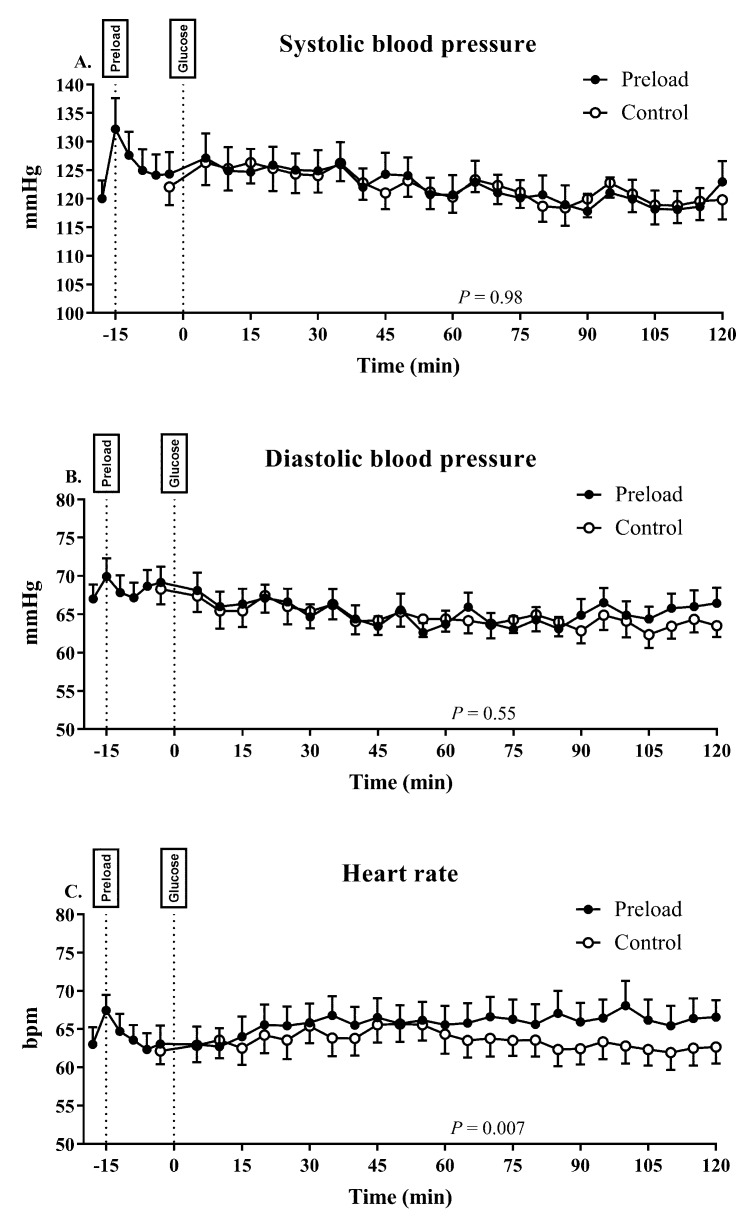
Systolic (**A**) and diastolic blood pressure (BP) (**B**) and heart rate (**C**) on the control and preload days. Data are mean values ± SEM (*n* = 18).

There was a modest fall in SBP on both days (*p* = 0.001 for both; Figure 4A), without any difference in the AUC0–120 min for SBP (*p* = 0.98), or the maximum fall in SBP (preload: −13.2 ± 2.5 versus control: −12.4 ± 1.7 mmHg; *p* = 0.77) between the two days.

#### 3.5.2. Diastolic Blood Pressure

There was a modest fall in DBP on both days (*p* < 0.01 for both; Figure 4B), without any difference in the AUC0–120 min for DBP (*p* = 0.55) or the maximum fall in DBP (preload: −11.4 ± 1.4 versus control: −11.1 ± 1.1 mmHg; *p* = 0.82) between the two days.

#### 3.5.3. Heart Rate

There was a modest increase in HR on both the preload (*p* = 0.001) and control (*p* = 0.009) days (Figure 4C). The AUC0–120 min for HR was greater (*p* = 0.007) on the preload day. The maximum rise in HR after the glucose drink also tended to be greater after the preload (preload: 11.3 ± 2.0 versus control: 7.9 ± 1.0 bpm, *p* = 0.08).

### 3.6. Relationships between Plasma Insulin and Serum 3-OMG between the Two Study Days

**Figure 5:**
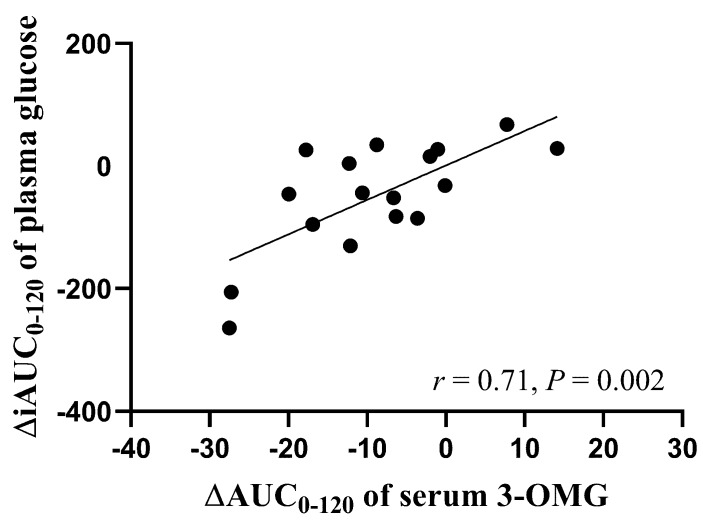
Relationship between the difference in incremental areas under the curves (iAUCs) for plasma glucose between the preload and control days and the difference in AUCs for serum 3-OMG between the preload and control days (*n* = 17).

Between *t* = 0 and 120 min, there was a correlation between the difference in iAUCs for plasma glucose and the difference in AUCs for serum 3-OMG between the preload and control days (*r* = 0.71, *p* = 0.002, Figure 5). However, there was no correlation between the difference in iAUCs for plasma glucose and the difference in iAUCs for plasma insulin between the preload and control days (*r* = 0.31, *p* = 0.21).

## 4. Discussion

Our study has shown that a whey protein/guar gum preload diminishes the glycaemic response to a glucose drink in healthy older subjects, as previously demonstrated in participants with T2D [1,2]. However, this effect was unrelated to a slowing of gastric emptying. There was a substantial delay in the absorption of glucose from the small intestine, and a stimulation, rather than a lowering, of insulin secretion. Moreover, changes in glycaemia and glucose absorption were related. The preload had no effect on the reduction in BP following the glucose load, but the latter was modest, and no subject had PPH. In contrast, the rise in SMA blood flow was attenuated by the preload.

The pivotal importance of upper gastrointestinal function to postprandial glycaemia in health and diabetes is now widely appreciated [34,35]. The focus has hitherto been on gastric emptying, which is frequently disordered in diabetes [36], albeit usually normal, or modestly accelerated, in uncomplicated T2D patients [7]. Postprandial glycaemic excursions are a major determinant of glycated haemoglobin, particularly when the latter is ≤8.0% [2], and can be diminished by slowing gastric emptying, which has stimulated the development of dietary and pharmacological strategies to target this mechanism. Examples of the latter are the amylin agonist, pramlintide [37], and ‘short-acting’ GLP-1 receptor agonists [38]. Our previous studies suggested that a whey/guar ‘preload’ reduced postprandial glycaemia in T2D predominantly by slowing gastric emptying [2,4]. However, the latter was assessed using a stable isotope breath test technique, which cannot discriminate effects of gastric emptying from those of small intestinal carbohydrate absorption. In the current study, the preload reduced glycaemia substantially, particularly given that healthy subjects were studied, rather than those with T2D, but it did not affect gastric emptying; indeed, the gastric emptying curves were essentially superimposed.

It should be appreciated that the preload (90 kcal) was taken 15 min before the drink, and because gastric emptying is usually in the range of 1–4 kcal/min [39,40], it would not be expected to have emptied completely from the stomach at the time of ingestion of the drink. Furthermore, at 120 min, the drink was not emptied from the stomach completely. Accordingly, we cannot exclude the possibility that, because of its higher intragastric caloric content, after the preload, while the rate of gastric emptying of the drink was unaffected between 0–120 min, the drink may have taken longer to empty completely. A reduction in small intestinal glucose absorption is shown unequivocally by the marked reduction in the rate of 3-OMG absorption in the absence of any difference in gastric emptying, and is accordingly, likely to contribute to glucose-lowering. This concept is also supported by the relationship observed between the changes in plasma glucose and 3-OMG. Guar has been shown to reduce small intestinal absorption of glucose [41,42] by forming a physical barrier between glucose and small intestinal mucosal cells [17,18]. In contrast to previous studies [2,43], the preload was associated with an increase in plasma insulin, which is likely to reflect the stimulation of insulin secretion by amino acids in the whey protein [4,5]. Slowing of gastric emptying has the capacity to override insulinotropic responses, as shown by the reduction in postprandial insulin induced by exogenous administration of GLP-1 [44,45].

Our study also evaluated the potential for the whey/guar preload in the management of PPH. Both oral (9 g) [18] and intraduodenal (4 g) [41] administration of guar diminished the hypotensive response to carbohydrates. There was, however, only a modest fall in BP in response to oral glucose, presumably because participants were all healthy, and no effect of the preload on BP. In contrast, the preload attenuated the rise in SMA blood flow substantially, presumably secondary to the reduction in glucose absorption. The small rise in HR after the preload might be attributable to the sympathetic effect of insulin [46,47]. Altogether, further studies in patients with PPH are needed before the approach is dismissed.

In interpreting our observations, specific limitations should be acknowledged: the sample size of the study population was relatively small; healthy older subjects rather than T2D patients were studied (as this was a study to assess mechanisms); and responses to oral glucose, rather than a meal, were evaluated. Given that none of our healthy older subjects experienced PPH, as stated above, we were unable to fully evaluate the therapeutic potential of the preload. Moreover, we did not measure GLP-1 or glucose-dependent insulinotropic polypeptide levels. The study was not placebo-controlled, as we hoped to capitalise on the potential beneficial effects of gastric distension on postprandial BP by the preload [13].

## 5. Conclusions

The reduction in the glycaemic response to oral glucose by a whey protein/guar gum preload in healthy older subjects reflects a slowing of small intestinal glucose absorption and a stimulation of insulin secretion, rather than a delay in gastric emptying.

## Figures and Tables

**Table 1:** Baseline (fasting) measurements in the subjects (*n* = 18) ^1^.

	Control (*t* = −3)	Preload (*t* = −18)	*p* Value
SBP (mmHg)	122.1 ± 3.2	120.0 ± 3.1	0.10
DBP (mmHg)	68.2 ± 1.9	67.3 ± 1.9	0.38
HR (beats/min)	62.1 ± 1.7	63.9 ± 2.3	0.09
Plasma glucose (mmol/L)	5.2 ± 0.1	5.2 ± 0.1	0.68
Plasma insulin (mU/L)	5.2 ± 0.9	5.3 ± 0.8	0.68
SMA blood flow (mL/min)	344 ± 23.6	329 ± 24.8	0.34

^1^ All values are absolute values. Differences between study days were tested via paired *t*-tests. Values are means ± SEM. SBP, systolic blood pressure; DBP, diastolic blood pressure; HR, heart rate; SMA, superior mesenteric artery.
